# Location Coding by Opponent Neural Populations in the Auditory Cortex

**DOI:** 10.1371/journal.pbio.0030078

**Published:** 2005-02-22

**Authors:** G. Christopher Stecker, Ian A Harrington, John C Middlebrooks

**Affiliations:** **1**Kresge Hearing Research InstituteUniversity of Michigan, Ann Arbor, MichiganUnited States of America; New York UniversityUnited States of America

## Abstract

Although the auditory cortex plays a necessary role in sound localization, physiological investigations in the cortex reveal inhomogeneous sampling of auditory space that is difficult to reconcile with localization behavior under the assumption of local spatial coding. Most neurons respond maximally to sounds located far to the left or right side, with few neurons tuned to the frontal midline. Paradoxically, psychophysical studies show optimal spatial acuity across the frontal midline. In this paper, we revisit the problem of inhomogeneous spatial sampling in three fields of cat auditory cortex. In each field, we confirm that neural responses tend to be greatest for lateral positions, but show the greatest modulation for near-midline source locations. Moreover, identification of source locations based on cortical responses shows sharp discrimination of left from right but relatively inaccurate discrimination of locations within each half of space. Motivated by these findings, we explore an opponent-process theory in which sound-source locations are represented by differences in the activity of two broadly tuned channels formed by contra- and ipsilaterally preferring neurons. Finally, we demonstrate a simple model, based on spike-count differences across cortical populations, that provides bias-free, level-invariant localization—and thus also a solution to the “binding problem” of associating spatial information with other nonspatial attributes of sounds.

## Abstract 2

A model relying on properties of auditory cortical neurons recorded in the cat can account for the accurate localization of sounds

## Introduction

Topographic representation is a hallmark of cortical organization: primary somatosensory cortex contains a somatotopic map of the body surface, primary visual cortex contains a retinotopic map of visual (retinal) space, and primary auditory cortex contains a cochleotopic map of sound frequency. The necessity of auditory cortex for normal sound localization (which is disrupted by cortical lesions [1,2,3]) strongly implies a cortical representation of auditory space. That representation has been reasonably expected to consist of a spatiotopic map, based on the existence of such maps in other sensory systems and on the view, proposed by Jeffress [4], that spatial processing in the auditory brainstem and midbrain might involve a “local code” consisting of topographic maps of interaural spatial cues. A local code, or “place code,” is one in which particular locations in space, or the spatial cues that correspond to those locations, are represented by neural activity at restricted locations in the brain. Evidence for local coding of auditory space has been demonstrated in mammalian superior colliculus [5,6] and in avian inferior colliculus (IC) [7,8] and optic tectum (homologous to mammalian superior colliculus) [9]. Nevertheless, local spatial coding has not thus far been demonstrated in the mammalian ascending auditory pathway.

If the Jeffress model is correct and a local code for spatial cues exists subcortically, one might anticipate local coding to be maintained in the cortex, where the various cues might finally be integrated into a coherent map of auditory space. Numerous studies, however, have failed to provide evidence for such a map. The spatial tuning of neurons is often characterized using rate–azimuth functions (RAFs), which specify the average response rate (spikes per trial or per second) as a function of stimulus location in the horizontal dimension. Throughout the auditory cortex, such functions typically exhibit broad peaks (up to 180° wide) that cover the contralateral hemifield and broaden further with increasing sound level [10,11,12,13,14]. Similar functions have been reported for cortical sensitivity to interaural cues [15,16], and for spatial and interaural sensitivity in the auditory brainstem and midbrain [17,18,19,20,21,22,23,24], thus questioning Jeffress's view of binaural processing in mammals. The emerging alternative view replaces the local code with a “distributed code,” in which sound-source locations are represented by patterns of activity across populations of broadly tuned neurons [12,24,25].

In the past, we argued for a distributed spatial code in the auditory cortex in part because the broad spatial tuning of cortical neurons would seem to preclude the existence of a local code and also because individual neurons are able to transmit spatial information throughout much, if not all, of auditory space [25,26]. At least implicitly, we have advocated a uniform distributed code, assuming that uniform sampling of space by RAF peaks is required for maximally accurate spatial coding. Spatial centroids of neurons in the posterior auditory field (PAF), for example, sample space more uniformly than neurons in the primary auditory field (A1), and we have suggested that this feature partially underlies the increased ability of ensembles of PAF neurons to accurately signal sound-source locations [14].

A number of observations demonstrate, however, that the auditory cortex samples space nonuniformly. RAFs are plotted for a selection of neurons in the dorsal zone (DZ) of auditory cortex in Figure 1, to illustrate a common observation of location-sensitive auditory cortical neurons: the majority favor contralateral stimulation, and typically exhibit either “hemifield” or “axial” tuning [11], responding to stimuli located throughout contralateral space or near the acoustic axis of the contralateral pinna, respectively. A smaller number of ipsilaterally tuned units are also observed, the majority of which exhibit hemifield or axial tuning characteristics similar to those of contralateral units. In A1 and DZ, ipsilateral- and/or midline-tuned neurons may be arranged in bands—parallel to the tonotopic axis—that interdigitate with bands of contralaterally tuned cells [25,27,28,29]. The overall preponderance of contralateral tuning among cortical units seems to justify the view that each hemisphere represents the contralateral spatial hemifield, a view that is also supported by the contralateral sound-localization deficits that follow auditory cortical lesions [2,30,31]. Even within a single hemifield, however, no strong evidence for a topographic representation has been reported, and the observation that many units share similar hemifield RAFs demonstrates a profound inhomogeneity in the way cortical populations sample auditory space.

**Figure 1:**
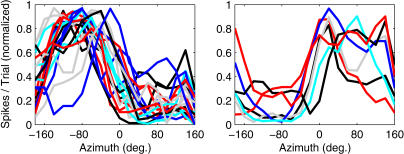
Example RAFs Plotted are normalized mean spike counts (y-axis) elicited by broadband stimuli (20 dB above unit threshold) varying in azimuth (x-axis). Lines represent units recorded in cortical area DZ. Left: contralaterally responsive units. Right: ipsilaterally responsive units.

Additional evidence that the cortical representation of auditory space is inhomogeneous comes from studies of the ability of cortical responses to classify stimulus locations. Stecker et al. [14] found that the responses of most spatially sensitive units in cat cortical areas A1 and PAF could accurately discriminate the lateral hemifield (left versus right) of a stimulus, but often confused locations within the hemifield. This is shown for six PAF neurons represented by confusion matrices in Figure 2. Similarly, Middlebrooks et al. [12] measured median localization errors—based on neural-network analyses of responses in the second auditory field (A2) and the field of the anterior ectosylvian sulcus (AES)—between 37.5° ± 8.9° and 43.7°*±* 10.2*°*, just under the theoretical limit of 45*°* attainable through perfect left/right discrimination and within-hemifield confusion. Taken together, these results suggest that auditory space is represented within the cortex by a population of broadly tuned neurons, each of which is able to indicate the lateral hemifield from which a sound originated, but generally little more.

**Figure 2:**
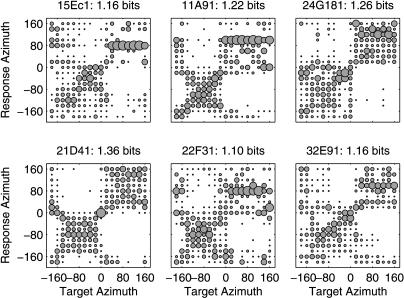
Classification Performance of Accurate PAF Units from [14] Neural spike patterns were classified according to the stimulus location most likely to have elicited them. In each panel, a confusion matrix plots the relative proportion of classifications of each target azimuth (x-axis) to each possible response azimuth (y-axis). Proportions are indicated by the area of a circle located at the intersection of target and response locations. Example units were selected from among those transmitting the most spatial information in their responses. In each case, discrimination of contralateral azimuths (negative values) from ipsilateral azimuths (positive values) is apparent, accompanied by significant within-hemifield confusion. As such, neural responses are sufficient for left/right discrimination only, and the spatial information transmitted by the most accurate units tends not to be much greater than one bit per stimulus.

## Results/Discussion

### Preferred Locations Oversample Lateral Regions of Contralateral Space—Steepest RAF Slopes Straddle the Midline

The idea that sound locations are signaled by the peaks of RAFs, which tend to be centered deep within the lateral hemifields, is at odds with localization behavior, which shows greatest resolution near the interaural midline [32,33]. An alternative view, however, has emerged for the processing of interaural time and level differences by cortical and subcortical neurons. In that view, locations are coded by the slopes, rather than the peaks, of rate–interaural-time-difference or rate–interaural-level-difference functions [22,24,34]. Moreover, these slopes appear aligned with the interaural midline and provide maximum spatial information in that region [20]. If a similar arrangement can explain the inhomogeneity of spatial sampling in the auditory cortex, then we would expect to find cortical RAF slopes to be steepest near the interaural midline as well.

In this report, we compare the responses of neurons in primary auditory cortex (A1) and two higher-order auditory cortical fields (PAF and DZ) in the cat. Compared to A1, areas PAF and DZ exhibit spectrotemporally complex responses that are significantly more sensitive to variations in sound-source location [14,25]. Therefore, these areas are the most likely candidate regions of cat auditory cortex for spatial specialization. PAF, in particular, appears necessary for sound localization by behaving cats [30].

Figure 3 depicts the distribution of preferred locations (“azimuth centroids”; see [14]) along with locations of peak RAF slopes in all three fields. As we have reported previously, centroid distributions in Figure 3 reveal a preponderance of contralateral sensitivity regardless of cortical area or stimulus level [14,25]. Distributions of peak-slope location, however, are tightly clustered around the frontal midline (median*±* standard error [see Materials and Methods] in A1, +15° ± 2.5°; in DZ, +5° ± 2.1°; in PAF, −5° ± 2.6°). Values in A1 fall significantly farther into the ipsilateral field than do those in DZ (*p* < 0.0004) or PAF (*p* < 0.0002), consistent with both the broader spatial tuning and less extreme azimuth centroids of A1 compared to PAF or DZ units [14,25]. Overall, the positioning of RAF slopes near the interaural midline suggests that auditory space is sampled inhomogeneously by the cortical population; the midline represents a transition region between locations eliciting responses from populations of contralateral- and ipsilateral-preferring units.

**Figure 3:**
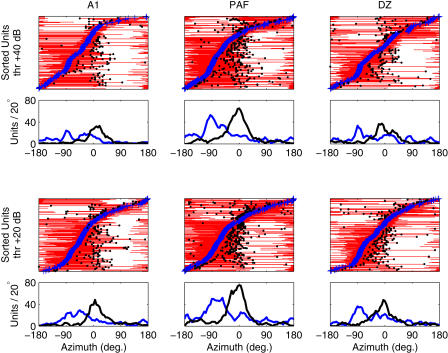
RAF Slopes Are Steepest near the Interaural Midline Plotted are summaries of preferred locations (centroids) and points of maximum RAF slope for 254 units recorded in A1 (left), 411 in PAF (middle), and 298 in DZ (right) for levels 20 and 40 dB above threshold (thr) (bottom and top rows, respectively). In each panel, units are sorted by centroid (blue crosses) on the y-axis. Thin red lines denote the region of azimuth (x-axis) containing the centroid and bounded by the points of steepest slope. For units with centroids lateralized more than 10° from the midline, we marked either the steepest positive slope (for ipsilaterally tuned units) or negative slope (for contralateral units) with a black circle. These points represent the location of most rapid response change that occurs toward the front of the animal (relative to the centroid; for units that respond throughout the frontal hemifield, this point can occur toward the rear). Distributions of centroid (blue line) and peak slope (black line), calculated using kernel density estimation with 20*°* rectangular bins, are plotted below each panel. These indicate that while preferred locations (centroids) are strongly biased toward contralateral azimuths, peak slopes are tightly packed about the interaural midline, consistent with the opponent-channel hypothesis.

### Neural Response Patterns Discriminate Best across Midline

Modulation of spike count is generally the most salient location-sensitive feature of neural responses, especially when data are averaged over many trials. However, temporal features of the neural response—such as first-spike latency, the temporal dispersion of spikes, and specific temporal features such as prototyped bursts of spikes or periods of inhibition—could also play an important role in stimulus coding by cortical neurons, and we have studied this role using pattern-recognition analyses applied to spike patterns [12,14,35]. Here, we assess the ability of neural spike patterns to subserve pairwise discrimination of stimulus locations by adapting the pattern-recognition approach of Stecker et al. [14] to a discrimination paradigm. This approach is similar to the receiver-operating-characteristic analysis used to estimate interaural and/or spatial thresholds from neural spike counts [36,37,38], with the addition of spike-timing information. Given a spike pattern—a smoothed, bootstrap-averaged peristimulus time histogram (2-ms bins) that approximates the instantaneous probability of spike firing over the course of 200 ms following stimulus onset—elicited by stimulation from an unknown location in space, the algorithm estimates the relative likelihood that the pattern was evoked by a sound from each of the 18 tested locations. From these relative likelihoods, we compute the index of discriminability, *d′* [39], for each pair of stimulus locations. In Figure 4 (right), pairwise *d′* is plotted as a function of the midpoint and separation between paired stimuli for a single PAF unit; the contour *d′* = 1 (dashed line) indicates the spatial discrimination threshold. Note that in this example suprathreshold discrimination is possible at much narrower stimulus separations when the stimuli span the interaural midline (left/right discrimination) than in cases of front/back discrimination spanning +/− 90*°*, about which point many features of the neural response (e.g., spike rate and latency) are symmetrical. As a result, the minimum discriminable angle (MDA, defined as the minimum separation along the *d′* = 1 contour) of 25*°* is found at a best azimuth (BA, the midpoint location of the most discriminable pair) of −5*°*, near the frontal midline.

**Figure 4:**
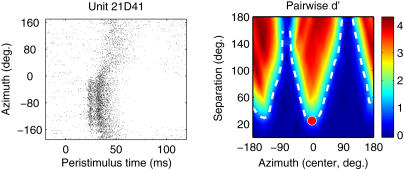
Discrimination Analysis Based on Responses of One PAF Unit Left: raster plot of spike times (x-axis) recorded in response to broadband noise stimuli varying in azimuth (y-axis). Note the strong modulation of spike count, response latency, and temporal features of the response between contralateral and ipsilateral locations. Right: pairwise spatial discrimination. Colors indicate *d′* values for pairs of stimulus locations varying in separation (y-axis) and overall azimuth (x-axis, midpoint of two azimuths). The dashed line indicates threshold discrimination (*d′* = 1), and the red circle marks the unit's MDA (y-axis) and BA (x-axis).

In Figure 5, MDA is plotted as a function of BA for the entire population of A1, PAF, and DZ units in which discrimination thresholds could be calculated. Overall, two main features of the results should be noted. First, despite the broad azimuth tuning of cortical neurons, the majority can discriminate between stimuli separated by less than 40*°*. A number of neurons successfully discriminate even smaller separations—especially in DZ, in which the median MDA (30.5*° ±* 2.5*°*) is significantly smaller than in A1 (40*° ±* 2.6°; *p* < 0.007) or PAF (43*° ±* 3.8*°*; *p* < 0.0002). Note that MDAs of even the most sensitive units exceed behavioral estimates of 5°–6*°* minimum audible angles in cats [40], but likely underestimate the true neuronal performance because loudspeaker separations were tested in minimum steps of 20*°*, and thus discrimination at smaller separations can only be assessed through extrapolation. Second, the distribution of BAs is tightly clustered around the interaural midline, with 50% of BA values falling within 18.5*°* (PAF), 25*°* (A1), or 26*°* (DZ) of the 0*°* or 180*°* azimuth. Note that this does not mean that units cannot discriminate off-midline locations. It does indicate, however, that the majority of units capable of discriminating between stimulus azimuths do so best for location pairs near the interaural midline. Very few units exhibit BAs located far within either lateral hemifield, although A1 units exhibit significantly more ipsilateral BA values (median, +14.5*° ±* 3.9*°*) than those in PAF (0*° ±* 2.9*°*; *p* < 0.004) or DZ (5*° ±* 4.3*°*; *p* < 0.05). As with the analysis of RAF slopes, the pairwise discrimination data reveal an inhomogeneous arrangement of spatial sampling by neurons in the cortical population. Accurate discrimination is found where RAF slopes are steepest (the midline), rather than where units respond most strongly (the lateral poles).

**Figure 5:**
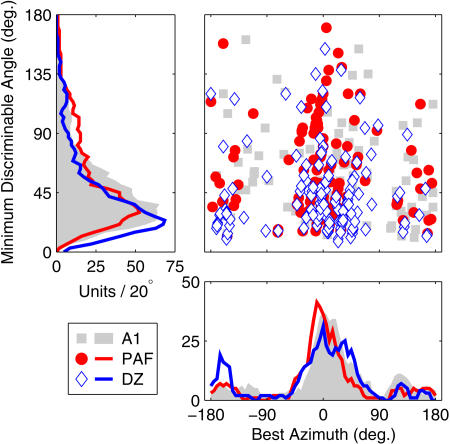
MDA by BA MDA (y-axis) is plotted against BA (x-axis) for each unit exhibiting suprathreshold spatial discrimination (see Materials and Methods). Symbols indicate the cortical area of each unit. Left and lower panels plot distributions of MDA and BA (in numbers of units per rectangular 20*°* bin), respectively.

### An Opponent-Channel Code for Auditory Space?

We have demonstrated quantitatively that the representation of auditory space in the cortex is inhomogeneous, consisting mainly of broadly tuned neurons whose responses change abruptly across the interaural midline. The population of auditory cortical neurons, then, appears to contain at least two subpopulations broadly responsive to contralateral and ipsilateral space. Neurons within each population exhibit similar spatial tuning and thus appear redundant with respect to spatial coding. The similarity of spatial tuning of units in these populations stands in contrast to their more profound differences in frequency tuning, for example. Each subpopulation, or “spatial channel,” is capable of representing locations on the slopes of their response areas (i.e., across the interaural midline) by graded changes in response—a “rate code” for azimuth, generalized to incorporate spatially informative temporal features of the cortical response [14,35,41]. In other words, each spatial channel encodes space more or less panoramically, as we have argued previously for individual cortical neurons [12], although it now seems clear that some regions of space are represented with greater precision than others. In the past, we have argued that auditory space is encoded by patterns of activation across populations of such panoramic neurons. Here, we amend that view—which remains tenable—to reflect the observed inhomogeneity of spatial sampling in the cortex and account for differences in coding accuracy of midline and other locations. Following the proposals of von Békésy [42] and van Bergeijk [43] regarding interaural coding in the brainstem, we propose that auditory space is encoded specifically by differences in the activity of two broad spatial channels corresponding to subpopulations of contralateral and ipsilateral units within each hemisphere (i.e., by a left/right opponent process). We will refer to this proposal as the opponent-channel theory of spatial coding in the auditory cortex.

An important consequence of the opponent-channel theory is that spatial coding may be robust in the face of changes in stimulus level. As is evident from past work, an important constraint on spatial coding in the cortex is the level dependence of many neurons' tuning widths, such that sharp tuning is seen predominantly for low-level stimulation. For example, a number of narrowly tuned units in Figure 3 exhibit locations of peak slope that closely track their centroids at 20 dB above threshold, and one could argue that such units form the basis of a local (e.g., topographic) spatial code when stimulus levels are low. Such a code, however, would be significantly impaired by increases in stimulus level—predicting that sound localization should be most accurate at low levels. That prediction is not borne out in psychophysical tests [44,45], and we have argued that spatial coding in the auditory cortex must employ relatively level-invariant features of the neural response [12]. Rather than relying on such features as they naturally occur, the opponent-channel mechanism constructs level-invariant features by comparing the activity of neurons that respond similarly to changes in level but differentially to changes in location, similarly to the coding of color by opponent-process cells in the visual system [46].

To illustrate the level invariance achieved by opponent-process coding, we analyzed the ability of cortical population responses to signal sound-source locations in the frontal hemifield under different stimulus-level conditions. The analysis (see Materials and Methods) is simplified in a number of ways—for example, it utilizes a simple linear decision rule that weights contralateral and ipsilateral input equally, sums across multiple neurons within each subpopulation (ignoring any complexity of neural circuitry), combines data across different cortical areas known to exhibit different spatial sensitivities, and reduces each neural response pattern to a single overall spike count—but serves as a “proof of concept” that differences between the responses of neural subpopulations with quasi-independent spatial tuning can be used to estimate sound-source locations in an unbiased manner when stimulus levels vary, whereas the individual population responses cannot.

Population responses (means of normalized spike rate across neurons in a population) to stimuli varying in location and level were computed separately for subpopulations composed of contralateral units or ipsilateral units in our sample of recordings in A1, PAF, and DZ. These subpopulations correspond to hypothetical “left” and “right” channels of a spatial coding mechanism. Classification of stimulus locations was based on either one of the subpopulation responses or the difference between the two, and involved linear matching to templates computed from a separate training set [14]. Population and difference RAFs are plotted in Figure 6 (left), along with confusion matrices (similar to Figure 2) for sound-source classification based on each (right). Relatively accurate classification is exhibited by both subpopulation responses and by their difference when test and training sets reflect the same stimulus level. When training and test sets differ, however, responses are systematically biased. After training with 20-dB stimuli, localization of 40-dB stimuli by the contralateral subpopulation is biased toward the contralateral hemifield, because 40-dB ipsilateral stimuli and 20-dB contralateral stimuli elicit similar responses. Similarly, when trained with 40-dB stimuli, localization of 20-dB stimuli is biased toward the ipsilateral hemifield. This pattern is clear in the responses of both the contralateral-preferring and ipsilateral-preferring subpopulations. Classification based on their difference, however, is relatively unbiased. Significant undershoot (central responses for peripheral stimulus locations) results from compression of population RAFs by intense sound. While we know of no behavioral data relating to the effects of stimulus level on sound localization by cats, undershoot has been reported in numerous studies of their localization behavior [47,48,49]. Such undershoots, however, need not be assumed to reflect a limitation of the underlying neural representation of auditory space.

**Figure 6:**
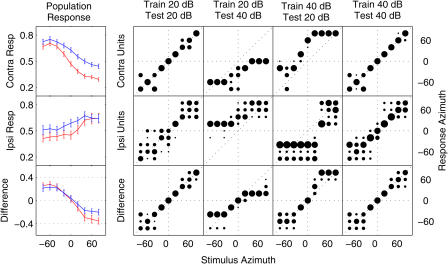
Difference between Channel Responses Is Less Sensitive to Changes in Level Than Are Channel Responses Themselves Left: population responses (y-axis; see Materials and Methods) are plotted as a function of azimuth (x-axis) for stimuli presented 20 dB (red) and 40 dB (blue) above unit thresholds. Population responses were computed separately for subpopulations composed of contralateral units (top) or ipsilateral units (middle) corresponding to hypothetical “left” and “right” channels of an opponent-channel spatial coding mechanism. The difference (bottom) between responses of the two subpopulations is more consistent across stimulus level than is either subpopulation response alone. Error bars indicate the standard deviation of responses across 120 simulated trials. Right: stimulus–response matrices (confusion matrices; see Figure 2) showing the proportion (area of black circle) of responses to a given (unknown) stimulus azimuth (x-axis) classified at each response azimuth (y-axis). Classification assigned each neural population response in the “test” set to the stimulus azimuth whose mean population response in an independently selected set of “training” trials was most similar. In some conditions, test and training trials were drawn from the same set of (matching level) trials: 20 dB (first column) or 40 dB (far right column). In others, test and training trials reflected different-level stimuli: 40-dB test stimuli classified based on a 20-dB training set (second column), or 20-dB test stimuli classified based on a 40-dB training set (third column). The contralateral and ipsilateral subpopulation responses (top and middle rows) accurately localize fixed-level stimuli, but are strongly biased when tested at non-trained stimulus levels. In contrast, the difference between responses (bottom row) remains relatively unbiased in all conditions, although responses to stimuli at untrained levels do exhibit compressed range and increased variability of classification.

Based on its manner of level-invariant spatial coding, it seems clear that an opponent-channel mechanism should behave similarly in the presence of any stimulus change (e.g., in frequency, modulation, or bandwidth) that acts to increase or decrease the response of both channels. This suggests an efficient means for combining spatial information with information about other stimulus dimensions. This general principle of opponent-process coding should hold in any case where both channels exhibit similar sensitivity to the nuisance dimension (level, frequency, etc.) but dissimilar sensitivity to space, and illustrates one strength of the opponent-channel coding strategy: the ability to recover spatial information from the responses of neurons that are strongly modulated by other stimulus dimensions. As long as some of the cortical neurons involved in coding a particular acoustic feature are contralaterally driven and others are ipsilaterally driven, the spatial location of that feature can be computed without imposing additional distortion of its neural representation.

Note that the opponent-channel theory as presented here involves contralateral and ipsilateral channels within each hemisphere. This feature is based on the observation of both types of neurons in a single hemisphere, and on the results of unilateral cortical lesions, which produce localization deficits mainly in contralesional space [2,30,31,50]. The lesion data prevent us from considering opponent-channel mechanisms that place each channel in a separate hemisphere (e.g., left-hemisphere contralateral units versus right-hemisphere contralateral units) because in that case unilateral lesions should abolish localization throughout the entire acoustic field. As proposed here, however, the opponent-channel mechanism in either hemisphere should be capable of coding locations throughout space, not just in the contralateral hemifield. This would suggest that only bilateral lesions could produce localization deficits, which is also not the case. At this point, we can merely speculate that auditory cortical structures in each hemisphere provide input only to those multimodal spatial or sensorimotor structures that subserve localization behavior in contralateral space and, furthermore, that these inputs cannot be modified in adulthood following cortical lesions.

### General Discussion

In summary, the available data suggest that space is sampled nonuniformly in all fields of auditory cortex, with the majority of neurons responding broadly within one hemifield and modulating their responses abruptly across the interaural midline. Consistent with this view, we found cortical responses to be most sensitive to changes in stimulus azimuth at midline locations. Cortical neurons' RAFs tend to be steepest near the midline even though their preferred locations are found distributed throughout the contralateral hemifield. Spatial discrimination by neural responses is also best at or near the interaural midline. Results of both analyses are compatible with the existence of a limited number of spatial channels in the cortex, and incompatible with either a uniform distributed representation or a local representation (e.g., a topographic map). The relative paucity of units with sharp tuning peaking near the midline strongly suggests that behavioral sound-localization acuity is mediated by the slopes and not the peaks of spatial receptive fields.

In this report, we consider a model of spatial coding based on differences in the response rates of two broad spatial channels in the auditory cortex. It is similar to the mechanism proposed by Boehnke and Phillips [51] to account for differences in human gap detection when gaps are bounded by auditory stimuli occurring in the same or opposite hemifields. In each proposal, neural response rates are compared across channels, but each is also consistent with information encoded in the relative response timing of cortical neurons [25,52]. Although the psychophysical and physiological data seem to agree on a two-channel mechanism, it is important to note that in this study, we treat units that respond more strongly to forward than rearward locations (“axial” units) as equivalent to units that respond equally to both quadrants (“hemifield” units). Similarly, we do not specifically examine the small number of units that respond best to midline locations. Distributed coding of interaural intensity by neural populations differing in binaural facilitation has been suggested previously [24]; similarly, populations of midline and/or axial units could be treated separately in a three-, four-, or five-channel opponent model of spatial coding. Such a model would follow the general principles of opponent-channel coding described here, but might differ in its ability to accurately code locations over wide regions of azimuth (see [24]).

That the representation of space appears inhomogeneous in both primary and higher-order auditory cortical fields argues against the existence of a topographic “space map” within sensory cortex, pushing the emergence of any such map further into central structures than previously expected. The processing of interaural cues begins at the level of the superior olivary complex, but the integration of such cues into a complete topographic map of auditory space is presumed to begin with processing at the level of the IC or cortex. The suggestion that interaural cues are represented by a limited number of binaural channels in the IC [22] seems to imply that the space map must emerge at the level of auditory cortex or beyond, and the results of this study, along with others [15,16], suggest that a “limited channel” code is maintained throughout primary and non-primary fields of the auditory cortex as well. PAF, in particular, appears to sit at the top of the auditory cortical processing hierarchy [53] but is similar to primary auditory cortex (A1) in this regard.

We should note that spatial coding must subserve at least two distinct behavioral tasks, namely, the discrimination of sound-source locations and the localization of individual sources (e.g., orientation, or pointing). Much of the current discussion has focused on aspects of spatial coding relevant to discrimination, and on the observation that the RAF slopes of cortical neurons are better suited to the discrimination of nearby locations than are their broad RAF peaks. Nevertheless, we are interested in general mechanisms of spatial representation, and argue that cortical neurons' broad spatial tuning suggests that neither aspect of sound localization is likely mediated by RAF peaks in cat cortex. This stands in contrast to the neural mechanism for sound localization in the IC of the owl, where sharp circumscribed spatial receptive fields form a place code for localization [7]. Owls' behavioral discrimination of spatial locations, however, is sharper than these neural receptive fields, and appears—as in mammals—to be mediated by receptive-field slopes [38]. Thus, the owl makes use of place and rate codes for different behavioral tasks. The cat's auditory cortex, on the other hand, lacks the sharp spatial tuning necessary for map-based localization, so one coding strategy underlies both types of behaviors.

It seems clear that these different coding strategies in owls and cats necessitate different mechanisms for generating motor responses and orienting to sound sources. The owl's space map exhibits a straightforward correspondence between restricted neural activity and locations in space, which might be ideal for computing audiovisual correspondence but requires further translation into motor coordinate systems before action can take place. It is possible that the opponent-channel code is transformed into a similar auditory space map within multisensory or sensorimotor areas, that is, not within auditory cortex itself. Alternatively, opponent-channel population codes in the auditory domain might be directly transformed into population codes in the motor domain without an intervening map-like representation. In either case, we could argue that the fundamental mode of spatial coding within the auditory system per se is non-topographic. In fact, it might be that auditory spatial topography is an emergent property of widespread neural populations and is evident only in perception and behavior, not in the physiology of single neurons.

In considering the relative advantages of opponent-channel spatial coding within the cortex, one might wonder whether the formation of a spatiotopic map would be necessary or desirable. As described above, the opponent-channel mechanism could subserve behavior without an intervening map, and it provides an efficient means of combining information about space with information about other stimulus features. In this regard, at least, the opponent-channel mechanism solves—or simply avoids—the so-called binding problem [54] of how multiple stimulus features can be associated to create a unified neural representation. It does so without recourse to specialized mechanisms for binding [55] and without an explosion in the number of neurons necessary for a complete combinatorial code [56]. So long as feature maps (e.g., of frequency) contain neurons of each class (i.e., contralateral and ipsilateral), the spatial position of any particular feature can be reconstructed without the difficulty of binding activity in one feature map (frequency) with that in another (location).

Finally, the three cortical fields studied in this report exhibited similar evidence for an opponent-channel mechanism, despite previously reported differences in their spatial sensitivity [14]. Although such differences appear modest when assessed physiologically, studies indicate that some fields are more critical for localization behavior than others [30]. An intriguing question for future research involves cortical fields—such as the anterior auditory field—that are not necessary for accurate localization. Are spatial channels maintained in such fields, or are they combined to produce space-invariant representations of other stimulus features?

## Materials and Methods

### 

Data analyzed for this report were collected from extracellular recordings of 254, 411, and 298 units in areas A1, PAF, and DZ (respectively) of the cortex of chloralose-anesthetized cats [14,25]. Methods of animal preparation, stimulus delivery, unit recording, and basic analysis have been described previously [14], and were approved by the University of Michigan Committee on Use and Care of Animals. Stimuli were delivered from loudspeakers placed in the free field, and consisted of 80-ms broadband noise bursts presented at levels 20–40 dB above unit threshold. Stimulus locations spanned 360*°* of azimuth in 20*°* steps, and are identified by angular distance from the frontal midline (0*°*). Positive azimuths increase to to the right (ipsilateral to the recording site), whereas negative values correspond to contralateral locations on the cat's left side. Unit activity was recorded extracellularly from the right cerebral hemisphere using 16-channel electrode arrays (“Michigan probes”), and spikes were sorted off-line based on principal-components analysis of their waveshapes.

#### Locations of peak slope and centroids

Each unit's preferred location was characterized by the azimuth centroid of response (dark blue crosses in Figure 3; see [14]); this is the spike-count-weighted average of contiguous stimulus locations eliciting a normalized response at or above 75% of maximum spike count per stimulus presentation. We additionally determined the locations of peak slope for each unit by smoothing its RAF (circular convolution with a 40*°* boxcar) and calculating the first spatial derivative of the result. Maximum and minimum values of the derivative indicate two peak-slope azimuths for each unit (black circles and endpoints of red horizontal lines in Figure 3).

#### Spatial discrimination by neural response patterns

Analyses of pairwise spatial discrimination (see Figures 4 and 5) employed a statistical pattern-recognition algorithm [14] to estimate the relative likelihood of each stimulus location, given the temporal pattern of neural response to a single (unknown) stimulus. We computed, for each pair of locations θ_1_ and θ_2_ in the loudspeaker array, the index of pairwise discriminability *d′* [39] based on the estimated relative likelihoods:



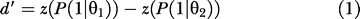



where *z*(*P*) represents scaling to the standard normal distribution and the probability *P* of responding “1” is given by the (estimated) relative likelihood *l* of location θ_1_ (versus θ_2_), conditional on the actual stimulus location θ*_i_*.



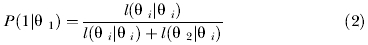



The analysis produces a map of *d′* between each pair of stimulus locations, plotted in coordinates of stimulus separation and overall location in Figure 4. The map was interpolated to find a contour of *d′* = 1, which we define as threshold discrimination. The smallest stimulus separation along the threshold contour defines the MDA, and the overall location of that stimulus pair defines the unit's BA. Symbols in Figures 4 and 5 indicate values of MDA and BA for individual units.

#### Evaluation of a simple population code for space

To assess the level invariance of opponent-channel coding, we analyzed a simplified model of population spatial coding in the cortex. For each neural unit in a channel (e.g., a subpopulation of contralateral-preferring units), we accumulated a list of responses (spike counts normalized to the maximum response across all trials) on each trial with a given combination of stimulus azimuth and level. Azimuths were confined to the frontal hemifield (−80° to +80°) to avoid front–back confusions, which obscure but do not alter the appearance of bias in classification responses, and levels were either 20 or 40 dB above individual unit thresholds. We then computed population responses by randomly selecting one trial (with matching stimulus azimuth and level) from each unit and computing the mean of individual responses. We repeated the selection process 120 times for each combination of azimuth and level to simulate a set of 120 population “trials.” The mean of these population responses for each stimulus is plotted on the left in Figure 6. Separate “training” and “test” sets of population responses were computed by this method and used to assess the ability of subpopulations to classify stimulus locations. Individual population responses in the test set were classified to the azimuth with the most-similar mean population response across the training set. Confusion matrices in Figure 6 plot the proportion of test-set responses assigned to each stimulus azimuth. In some conditions, test and training sets were drawn from the same trials (matching level); in other conditions, training and test sets differed in stimulus level.

We tested classification based on responses of a contralateral subpopulation, an ipsilateral subpopulation, and on the difference between subpopulation responses. Contralateral and ipsilateral subpopulations were composed of all units with centroids falling farther than 30° into the corresponding hemifield in our sample of A1, PAF, and DZ units. Differences were computed from the two subpopulation responses on a trial-by-trial basis, and classification was tested in the same manner as for the population responses themselves.

#### Statistical procedures

Tests of statistical significance in this study were conducted using a 5,000-permutation bootstrap test (see [14] for details), reported to one significant digit. Standard error of the median, where reported, was obtained using a 2,000-permutation bootstrap, drawing *N* (the total number of data points) samples from the data with replacement on each permutation and recomputing the median. Distributions in Figures 3 and 5 were computed by kernel density estimation (convolution) with a 20*°* rectangular window to obtain a continuous function of units per 20*°* bin.

## Abbreviations

A1: primary auditory field
BA: best azimuth
DZ: dorsal zone
IC: inferior colliculus
MDA: minimum discriminable angle
PAF: posterior auditory field
RAF: rate–azimuth function
